# The Prevalence of Anticholinergic Drugs and Correlation with Pneumonia in Elderly Patients: A Population-Based Study in Taiwan

**DOI:** 10.3390/ijerph17176260

**Published:** 2020-08-28

**Authors:** Chien-Ying Lee, Yih-Dih Cheng, Wei-Yuan Cheng, Tung-Han Tsai, Kuang-Hua Huang

**Affiliations:** 1 Department of Pharmacology, Chung Shan Medical University, Taichung 40201, Taiwan; cshd015@csmu.edu.tw; 2 Department of Pharmacy, Chung Shan Medical University Hospital, Taichung 40201, Taiwan; 3 School of pharmacy, China Medical University, Taichung 40402, Taiwan; tovis168@gmail.com; 4 Department of Pharmacy, China Medical University Hospital, Taichung 40402, Taiwan; 5 Taso-Tun Psychiatric Center, Ministry of Health and Welfare, Nantou 54249, Taiwan; continentaln78005@gmail.com; 6 Department of Health Services Administration, China Medical University, Taichung 40402, Taiwan; dondon0525@gmail.com

**Keywords:** anticholinergic drugs, pneumonia, elderly, potentially inappropriate medication, pharmacoepidemiology

## Abstract

Anticholinergic drugs may increase the risk of serious respiratory infection, especially in the elderly. The study aims to investigate the prevalence of anticholinergic drugs and the correlation of incident pneumonia associated with the use of anticholinergic drugs among the elderly in Taiwan. The study population was 275,005 elderly patients aged ≥65 years old, selected from the longitudinal health insurance database (LHID) in 2016. Among all the elderly patients, about 60% had received anticholinergic medication at least once. Furthermore, the study selected elderly patients who had not been diagnosed with pneumonia and had not received any anticholinergic drugs in the past year in order to evaluate the correlation between pneumonia and anticholinergic drugs. The study excluded elderly patients who died or had received related drugs of incident pneumonia during the study period and selected elderly patients receiving anticholinergic drugs as the case group. Propensity score matching (PSM) on a 1:1 scale was used to match elderly patients that were not receiving any anticholinergic drugs as the control group, resulting in a final sample of 32,215 patients receiving anticholinergic drugs and 32,215 patients not receiving any anticholinergic drugs. Conditional logistic regression was used to estimate the association between anticholinergic drugs and pneumonia after controlling for potential confounders. Compared with patients not receiving anticholinergic drugs, the adjusted odds ratio of patients receiving anticholinergic drugs was 1.33 (95% confidence interval: 1.18 to 1.49). Anticholinergic medication is common among elderly patients in Taiwan. Elderly patients receiving anticholinergic drugs may increase their risk of incident pneumonia. The safety of anticholinergic drugs in the elderly should be of concern in Taiwan.

## 1. Introduction

Anticholinergic drugs act centrally and peripherally on muscarinic acetylcholine receptors. Cimetidine, metoclopramide, and ranitidine are commonly employed in the treatment of the urinary bladder, the respiratory tract, and gastroesophageal disease via the intervention of the muscarinic receptor [1]. The peripheral anticholinergic complaint of dry mouth can promote mucosal damage and increase the risk for serious respiratory infection, secondary to losing the effect of the antimicrobial activity of saliva [2]. Owing to the nonselective nature of this muscarinic receptor antagonist, elderly patients are particularly susceptible to anticholinergic drugs because of age-related changes in pharmacokinetic and pharmacodynamic properties [3].

Anticholinergic drugs are one of the risk factors for pneumonia in elderly patients. It can increase the risk of pneumonia in elderly patients, although the strength of the muscarinic blockade of every anticholinergic drug is different [4,5]. One of the mechanisms for pneumonia is that dry mouth caused by anticholinergic drugs may lead to oropharyngeal and esophageal swallowing impairments and result in aspiration pneumonia [6,7]. The impairment of airway mucociliary transport in patients can prolong bacterial stay in the lungs and eventually contribute to respiratory infections; in addition, lower esophageal sphincter pressure can lead to acid reflux and cause aspiration [8]. The central effects of anticholinergic drugs may increase the risk of pharyngeal aspiration, including sedation and altered mental status, then lead to the incident pneumonia. This can more likely result in bacterial pneumonia when aspirated bacteria are not effectively cleared [9,10]. Sedation and altered mental status have also been associated with poor pulmonary hygiene and may lead to viral respiratory infection. It may also contribute to pneumonia [11]. Another mechanism that could be involved in anticholinergic-induced pneumonia is low levels of mucous secretions, which may increase the risk of incident pneumonia because of bacterial growth [6]. Previous literature suggests that oropharyngeal aspiration is a prominent etiologic factor for pneumonia in elderly adults with swallowing disorders and weak cough reflexes [9,10,11].

Many elderly patients suffer from multiple chronic diseases and commonly take different prescription medications for multiple conditions. It is known that changes in drug metabolism in the elderly occur with age. There is a common finding in elderly patients, where the use of multiple drugs may cause serious side effects. Many age-related diseases and disease-related conditions may predispose geriatric patients to anticholinergic intoxication [2]. In clinical practice, medications with anticholinergic effects are considered potentially inappropriate when used on the elderly [12,13]. Consequently, anticholinergic rating scales (ARS) are used to express the grade of anticholinergic effect in clinical practice, ranging from limited or none (Level 0), moderate (Level 1), strong (Level 2), and very strong (Level 3) [14,15], while the correlation between anticholinergic gradation and incident pneumonia is inconsistent. Some studies have indicated that the risk of incident pneumonia is not related to high potency anticholinergic drugs (Level 3) [6]. Previous studies have identified several drugs with anticholinergic properties we should be aware of, namely, the possibility of side effects in elderly patients, including respiratory system medicines (cetirizine, loratadine, pseudoephedrine), psychotropic medicines (paroxetine, quetiapine), alimentary tract medicines (cimetidine, ranitidine), and neurological disorder medications (larbidopa–levodopa, levodopa) [15,16,17].

To date, few studies have been conducted using a nationwide database to evaluate the correlation between pneumonia and anticholinergic drugs in elderly patients, especially in Taiwan [6,18]. It is essential to understand the risk of pneumonia in elderly patients receiving anticholinergic drugs. Therefore, this study was conducted to investigate the prevalence of anticholinergic drugs and the risk factors related to pneumonia and, moreover, estimate the correlation of incident pneumonia associated with the use of anticholinergic drugs among the elderly by using a nationwide database in Taiwan.

## 2. Materials and Methods

### 2.1. Data Source

The study is secondary data analysis, from 2015 to 2016, based on the longitudinal health insurance database (LHID) released by the Health and Welfare Data Science Center, Ministry of Health and Welfare (HWDC, MOHW; Registration No. H107175). LHID randomly selected two million beneficiaries from the Taiwan National Health Insurance (NHI) program. The information in LHID included detailed clinical records of the outpatient department and hospitalization, diagnostic codes, and prescribing information. The NHI program is nationwide social insurance that has enrolled up to 99% of citizens since 1995. Hence, the database is a nationally representative health database for Taiwan. HWDC provides scrambled random identification numbers for insured patients to protect the privacy of beneficiaries. This study protocol was approved as a completely ethical review by the Institutional Review Board of China Medical University Hospital, Taiwan (No. CMUH107-REC2-004). The database is anonymous; therefore, the requirement for informed consent was waived.

### 2.2. Study Subjects

**Figure 1:**
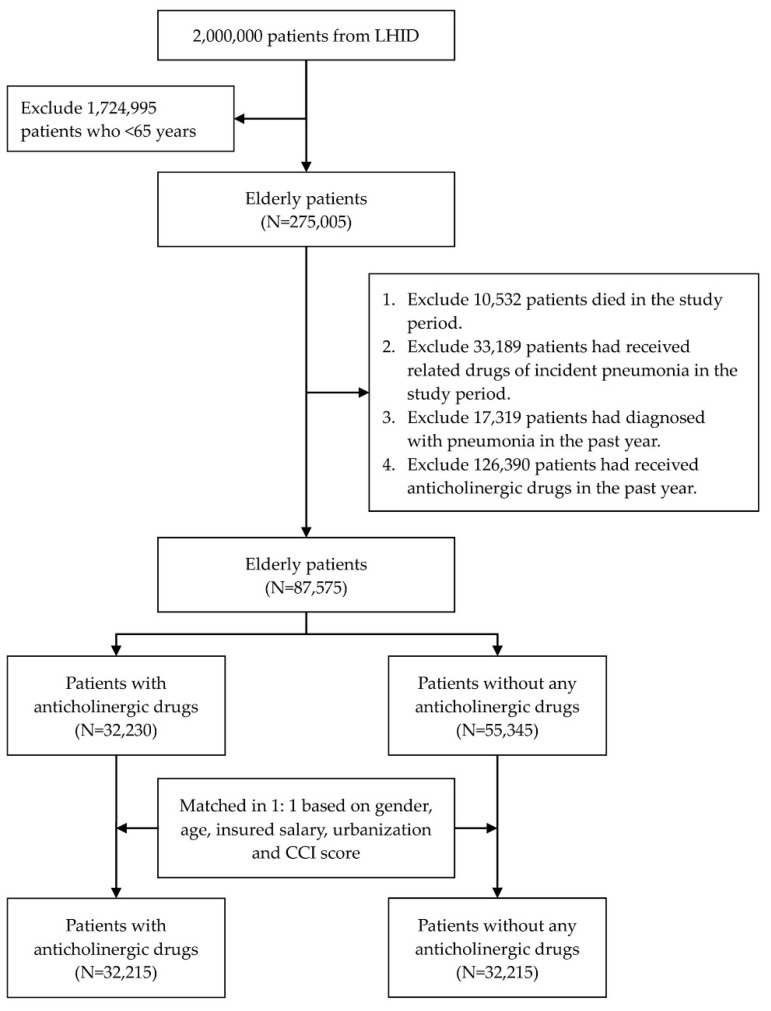
Flowchart of the study subject selection process. (Abbreviations: LHID, longitudinal health insurance database; CCI, Charlson comorbidity index)

Figure 1 lists the flowchart of the selected patients for inclusion. The study population was 275,005 elderly patients aged ≥65 years old, selected from LHID on 1 January 2016 to investigate the prevalence of anticholinergic drugs. Furthermore, the elderly patients who had not been diagnosed with pneumonia and had not received any anticholinergic drugs in the past year were enrolled in the study to evaluate the correlation between pneumonia and anticholinergic drugs. We also excluded 10,532 patients who died and the 33,189 patients who had received related drugs of incident pneumonia in the study period in order to improve the validity of the study results. The related drugs of incident pneumonia contained amiodarone, angiotensin-converting enzyme inhibitors (ACEIs), amantadine, and steroids [12,13,14,15,16,17]. Moreover, the study classified elderly patients according to their medication records in 2016 and selected elderly patients who were receiving anticholinergic drugs as the case group. In the study, there were a total of 49 drugs with the potential to cause anticholinergic adverse effects [17], including psychotropic medicines (e.g., paroxetine, quetiapine, trazodone), alimentary tract and metabolism medicines (e.g., cimetidine, metoclopramide, ranitidine, loperamide), respiratory system and allergy medicines (e.g., cetirizine, loratadine, pseudoephedrine), and neurological disorder medications (e.g., carbidopa–levodopa, levodopa) (see Table A1). Furthermore, to reduce the potential confounding caused by unbalanced covariates in nonexperimental settings, we used propensity score matching (PSM) on a 1:1 scale to match elderly patients not receiving any anticholinergic drugs as the control group, resulting in a final sample of 32,215 patients receiving anticholinergic drugs and 32,215 patients not receiving any anticholinergic drugs. The propensity score of the study is the probability of patients receiving anticholinergic drugs, calculated by gender, age, insured salary, urbanization, and the Charlson comorbidity index (CCI).

### 2.3. Study Design

**Figure 2:**
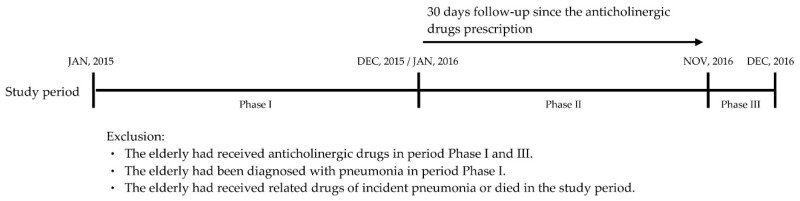
The study design diagram.

The study is a retrospective study to investigate the prevalence of anticholinergic drugs among elderly patients in Taiwan. Furthermore, the study design is divided into three phases to assess the correlation between anticholinergic drugs and pneumonia. The first phase was the exclusion period, from 1 January to 31 December 2015, in which to select the study subjects. The second phase was the observation period, from 1 January to 30 November 2016, in which to estimate the correlation between anticholinergic drugs and pneumonia. Patients were followed-up 30 days after anticholinergic drug prescriptions in order to observe the incidence of pneumonia. The third phase was in December 2016 to ensure the follow-up period. Thus, the patients receiving anticholinergic drugs in this phase would not be enrolled as study subjects. The study design diagram is exhibited in Figure 2.

The definition of incident pneumonia in the study was according to the principal diagnosis code in J12–J18, based on the International Classification of Diseases, Tenth Revision, Clinical Modification (ICD-10-CM). The study investigated the association between anticholinergic drugs and pneumonia via conditional logistic regression. Control variables in the study contained gender, age, insured salary, urbanization, CCI scores, and comorbidities related to pneumonia. Comorbidities contained diabetes mellitus (DM; ICD-10-CM: E08-E13), Alzheimer’s disease (AD; ICD-10-CM: G30, F00), stroke (ICD-10-CM: I60-I69), Parkinson’s disease (PD; ICD-10-CM: G20), major depression disorder (MDD; ICD-10-CM: F32.9, F33.9), chronic kidney disease (CKD; ICD-10-CM: N18), asthma (ICD-10-CM: J45), chronic obstructive pulmonary disease (COPD; ICD-10-CM: J40-J44, J47), heart failure (HF; ICD-10-CM: I50), upper respiratory infection (URI; ICD-10-CM: J00-J06), gastroesophageal reflux disease (GERD; ICD-10-CM: K21), and epilepsy (ICD-10-CM: G40-G41). The definition of comorbidities was with diagnosis at least three times a year, except epileptic seizure. Epileptic seizure was defined by diagnosis once a year.

### 2.4. Statistical Analysis

Descriptive statistics were used to summarize distributions of the prevalence of anticholinergic drugs in the elderly in Taiwan. We used the standardized mean difference to examine the balance of case and control groups after matching. We further used conditional logistic regression to estimate the association between anticholinergic drugs and pneumonia after controlling for potential confounders. SAS software version 9.4 (SAS Institute Inc., Cary, NC, USA) was used for statistical analysis in the study, and the statistical significance was defined as *p*-value < 0.05.

## 3. Results

### 3.1. The Prevalence of Anticholinergic Drugs

Table 1 presents the prevalence of anticholinergic drugs in elderly patients. There was a total of 967,887 prescriptions with anticholinergic drugs, about 12.96%, out of all prescriptions. The most frequency anticholinergic prescriptions were cimetidine (2.54%), cetirizine (1.19%), pseudoephedrine (1.15%), metoclopramide (1.13%), ranitidine (0.83%), quetiapine (0.80%), carbidopa–levodopa (0.66%), loratadine (0.65%), trazodone (0.54%), and loperamide (0.51%), sequentially. To elderly patients, there were 165,262 patients (60.09%) who had received anticholinergic medication at least once out of all elderly patients.

### 3.2. The Baseline Characteristics Distribution of Study Subjects

After subject selection, there was a total of 64,430 elderly patients in the study, and both groups, with and without anticholinergic drugs, had 32,215 patients. The mean age of patients receiving anticholinergic drugs was 73.58 ± 7.35 years, 51.77% were female, and 72.74% were in highly urbanization areas. As in Table 2, baseline characteristics were well balanced between elderly patients with and without anticholinergic drugs after matching. Among patients receiving anticholinergic drugs, there were 8614 patients (26.74%) with DM, 3457 patients (10.73%) with stroke, 317 patients with Parkinson’s disease (0.98%), 3684 patients with CKD (11.43%), 1928 patients with asthma (5.98%), 2781 patients with COPD (8.63%), 1076 patients with HF (3.34%), 18,688 patients with URI (58.01%), 862 patients with GERD (2.68%), and 120 patients with epilepsy (0.37%).

### 3.3. The Incidence Rate of Pneumonia in Elderly Patients

Table 3 indicates that among all participants, 1519 patients had occurred pneumonia, and the incidence rate was 23.58 cases per 1000 elderly patients. The incidence rate of patients receiving anticholinergic drugs was 33.23 cases per 1000 elderly patients. Compared with patients not receiving anticholinergic drugs, the risk ratio was 2.18 times of incident pneumonia. Compared with patients without DM, patients with DM had 1.31 times the risk of incident pneumonia. Additionally, compared with patients without comorbidities, patients with comorbidities had a higher risk of incident pneumonia, including stroke (2.21 times), PD (2.89 times), CKD (1.85 times), asthma (5.54 times), COPD (6.77 times), HF (2.75 times), URI (1.98 times), GERD (2.15 times), and epilepsy (4.61 times).

### 3.4. Correlation between Anticholinergic Drugs and Pneumonia

Table 4 points out that patients receiving anticholinergic drugs increased their risk of incident pneumonia (adjusted odds ratio [OR]: 1.33; 95% confidence interval [CI]: 1.18 to 1.49) after controlling for potential confounders. DM increased the risk of incident pneumonia (aOR: 1.44; 95% CI: 1.26 to 1.65). AD also increased the risk of incident pneumonia, but had no statistical significance (aOR: 1.20; 95% CI: 0.66 to 2.17). aORs of incident pneumonia in patients with comorbidities were all significantly higher than patients without, including stroke (aOR: 2.02; 95% CI: 1.75 to 2.33), PD (aOR: 1.94; 95% CI: 1.28 to 2.94), CKD (aOR: 1.57; 95% CI: 1.35 to 1.82), asthma (aOR: 1.57; 95% CI: 1.31 to 1.87), COPD (aOR: 4.93; 95% CI: 4.21 to 5.78), HF (aOR: 1.64; 95% CI: 1.32 to 2.04), URI (aOR: 1.79; 95% CI: 1.60 to 2.00), GERD (aOR: 1.52; 95% CI: 1.15 to 2.01), and epilepsy (aOR: 3.11; 95% CI: 1.92 to 5.03).

## 4. Discussion

Our study results indicate that about 60% of elderly patients had received anticholinergic medication at least once, and the most frequent anticholinergic prescriptions in Taiwan were cimetidine, cetirizine, pseudoephedrine, metoclopramide, and ranitidine. These drugs are used to treat URI and excessive secretions of gastric acid in primary care clinics in Taiwan. A previous study indicated that most frequent anticholinergic drugs were ranitidine, trazodone, paroxetine, oxybutynin, and nortriptyline [19]. Those were used to treat cardiovascular and neurological disorders. This indicates that there is a wide disparity in the prescription pattern of anticholinergic drugs between other countries and Taiwan. It may be related to the difference in medical care-seeking behavior caused by the health care system.

Pneumonia is a life-threatening infectious disease where age is one of the strongest risk factors. Our study demonstrates that anticholinergic drugs are related to incident pneumonia in the elderly. A population-based case–control study by Paul et al. [10] expressed that anticholinergic medication use is associated with pneumonia risk, compared with no use. Moreover, both acute and chronic use of anticholinergic drugs were associated with a higher risk for pneumonia, whereas there was no association with previous use. Another case–control study in America by Chatterjee et al. [6] estimated anticholinergic drug exposure and the risk of pneumonia. The overall use of anticholinergic drugs was associated with the risk of pneumonia, while the effect of anticholinergic drugs was not related to pneumonia. It indicated that even low-potency anticholinergic drugs may cause a significant risk of pneumonia. A nationwide study in Finland had a similar result [18]. Most of the low-potency anticholinergic drugs are cardiovascular drugs, and the risk may be partially explained by comorbidity. The main purpose of our study was to explore the potential relationship between the risk of pneumonia and anticholinergic drug use in the elderly in Taiwan. To enhance the accuracy of the study results, our study used PSM to obtain control groups for each elderly patient receiving anticholinergic drugs. A propensity score is a unit with certain characteristics that is assigned to each elderly patient receiving anticholinergic drugs. The scores can be used to reduce or eliminate selection bias in observational studies by the characteristics of elderly patients with and without anticholinergic drugs [20,21,22,23]. Compared with previous studies, our study design and methodology are more rigorous. Our study was inappropriate for further analysis regarding the effect of different levels of anticholinergic drugs due to the elderly subjects in our study being matched based on whether they received anticholinergic drugs rather than different levels of anticholinergic drugs. To our knowledge, there has been no related population-based study to estimate the relationship between incident pneumonia and anticholinergic drug use in Taiwan. Our study was not aimed at exploring the mechanisms between anticholinergic drugs and increased pneumonia risk, but the association between anticholinergic drugs and pneumonia in the elderly was clear. Although the incident rate of pneumonia caused by anticholinergic drugs is relatively rare, future studies should develop more well-designed prospective population trials to be carried out to ascertain the relationship.

The comorbidities may also predispose elderly patients to an increased risk of pneumonia. Our study results were consistent with previous studies that noted that comorbidities, including DM, stroke, PD, CKD, asthma, COPD, HF, URI, and GERD, were risk factors of pneumonia. The elderly with COPD or asthma are a high-risk population of bacterial infection, and one of the major infections is caused by streptococcus pneumoniae bacteria. It can cause many types of illnesses that contained pneumonia, meningitis, and septicemia. Therefore, COPD and asthma are both associated with a greater risk of incident pneumonia in the elderly [24,25,26]. DM may also increase the pneumonia risk in the elderly due to poor glycemic control. Aging is associated with a progressive decline in respiratory system function, and poor glycemic control can cause microvascular complications of lung capillaries [27,28,29]. Moreover, previous studies have documented that the elderly with stroke, dementia, or Parkinson’s disease have a higher risk of incident pneumonia [26,27,30]. It was possibly attributable to dysphagia, difficulty swallowing, and cough reflexes. Oropharyngeal dysphagia is also one of the risk factors for pneumonia in elderly patients [31,32,33]. Renal disease in the elderly is clinically important. Previous studies have described that there is a positive direction in the association between predialysis CKD and acute community-acquired infection [34]. Hypoimmunity of the elderly patient with CKD may predispose the patient to acute lower respiratory infections. Another study demonstrated that cardiovascular function declined in elderly patients with heart disease, which would affect the mucociliary clearance functions that trap and remove particulates and pathogens from the airways, leading to an increased risk of incident pneumonia [35]. Furthermore, a population-based cohort study indicated that GERD is associated with a long-term risk of pneumonia, especially in GERD with proton pump inhibitors (PPIs) for a longer treatment than four months [36]. There is a relationship between gastric acid suppressants and an increased risk of pneumonia [37]. Authors have suggested that the inhibition of gastric acid secretion by acid-suppressive therapy allows pathogen colonization from the upper gastrointestinal tract [24].

The major strength of the study is that it is based on a nationwide database, avoiding bias such as selection, nonresponse, and poor recall. Big data analysis is a new trend in modern healthcare research. LHID has completeness in recording prescriptions and clinical diagnosis. Moreover, the study was not only adjusted for potential confounding factors but also used PSM to avoid bias in the selection of study subjects. Therefore, the study indicates the correlation between anticholinergic drugs and pneumonia with a narrower and statistically significant confidence interval.

There were also a few limitations to the study. Some pneumonia-related variables, such as medication adherence, tobacco consumption behavior, and laboratory parameters, cannot be obtained from LHID. Additionally, the study only used the ICD code to define disease without any medical procedure codes. There may be overdiagnosis. Although our study indicated the correlation between anticholinergic drugs and pneumonia, the strength of the anticholinergic effect may also be related to pneumonia. We will explore the risk of incident pneumonia with different anticholinergic drugs in further study. Finally, this study is a type of observational study that analyzes data from a population database. The study result can only provide evidence to demonstrate that anticholinergic drugs are related to incident pneumonia. It is essential to obtain more information from other databases to analyze the cause–effect relation in future research.

## 5. Conclusions

The use of anticholinergic drugs is common in the elderly in Taiwan because these medications are prescribed for the symptomatic management of medical conditions. Elderly patients receiving anticholinergic drugs may increase their risk of incident pneumonia. Moreover, DM, stroke, PD, CKD, asthma, COPD, HF, URI, and GERD were also associated with incident pneumonia in the elderly. Anticholinergic drugs can have many beneficial effects, but these drugs need to be balanced against potential harm. Drugs with anticholinergic properties can be problematic, especially for the elderly population. Prescribers should consider dose reductions and monitoring when prescribing anticholinergic drugs to elderly patients to reduce the risks of adverse outcomes. Furthermore, randomized clinical trials are warranted to determine the effectiveness and safety of anticholinergic drugs in the elderly.

## Figures and Tables

**Table 1:** The distribution of prescriptions in elderly patients.

Prescriptions	No. of Prescriptions		No. of Patients Had Received	
	N	%	N	%
Total	7,467,908	100.00	275,005	100.00
Without anticholinergic drugs	6,500,021	87.04	109,743	39.91
With anticholinergic drugs	967,887	12.96	165,262	60.09
Most frequency anticholinergic drugs				
Cimetidine	189,862	2.54	53,171	19.33
Cetirizine	88,587	1.19	33,987	12.36
Pseudoephedrine	86,201	1.15	36,453	13.26
Metoclopramide	84,483	1.13	30,241	11.00
Ranitidine	61,831	0.83	16,066	5.84
Quetiapine	59,961	0.80	8126	2.95
Carbidopa–Levodopa	49,145	0.66	6371	2.32
Loratadine	48,793	0.65	20,132	7.32
Trazodone	40,079	0.54	6875	2.50
Loperamide	38,237	0.51	20,527	7.46

**Table 2:** The baseline characteristics distribution of study subjects after matching.

Variables	Crude Data						After Matching					
	Anticholinergic Drugs				SMD ^3^	*p*-Value ^1^	Anticholinergic Drugs				SMD ^3^	*p*-Value ^1^
	Without		With				Without		With			
	N	%	N	%			N	%	N	%		
Total	55,345	100.00	32,230	100.00			32,215	100.00	32,215	100.00		
Gender ^2^					0.08	<0.001					0	0.969
Female	27,610	49.89	17,337	53.79			17,317	53.75	17,322	53.77		
Male	27,735	50.11	14,893	46.21			14,898	46.25	14,893	46.23		
Age (year) ^2^	73.28±7.42		73.58±7.35		0.05	<0.001	73.59±7.42		73.58±7.35		0	0.981
65–70	25,432	45.95	14,034	43.54			13,992	43.43	14,034	43.56		
71–75	11,619	20.99	6881	21.35			6878	21.35	6878	21.35		
76–80	8317	15.03	5322	16.51			5344	16.59	5310	16.48		
>80	9977	18.03	5993	18.59			6001	18.63	5993	18.60		
Insured salary ^3^					0.06	<0.001					0	0.994
<20,008	18,342	33.14	9070	28.14			9046	28.08	9070	28.15		
20,008–22,800	19,850	35.87	13,174	40.87			13,184	40.93	13,159	40.85		
22,801–50,600	11,176	20.19	6632	20.58			6621	20.55	6632	20.59		
>50,600	5977	10.80	3354	10.41			3364	10.44	3354	10.41		
Urbanization ^2^					0.07	<0.001					0	0.903
Urban	42,143	76.15	23,434	72.71			23,480	72.89	23,434	72.74		
Suburban	8781	15.87	5759	17.87			5729	17.78	5747	17.84		
Rural	4421	7.99	3037	9.42			3006	9.33	3034	9.42		
CCI ^2, 3^					0.22	<0.001					0	0.989
0	32,820	59.30	15,617	48.45			15,626	48.51	15,617	48.48		
1–2	18,210	32.90	13,155	40.82			13,138	40.78	13,155	40.84		
≥3	4315	7.80	3458	10.73			3451	10.71	3443	10.69		
DM ^3^					−0.14	<0.001					−0.02	0.015
No	43,788	79.12	23,605	73.24			23,872	74.10	23,601	73.26		
Yes	11,557	20.88	8625	26.76			8343	25.90	8614	26.74		
AD ^3^					−0.04	<0.001					−0.03	<0.001
No	55,148	99.64	32,012	99.32			32,076	99.57	31,997	99.32		
Yes	197	0.36	218	0.68			139	0.43	218	0.68		
Stroke					−0.11	<0.001					−0.04	<0.001
No	51,081	92.30	28,768	89.26			29,131	90.43	28,758	89.27		
Yes	4264	7.70	3462	10.74			3084	9.57	3457	10.73		
PD ^3^					−0.08	<0.001					−0.08	<0.001
No	55,175	99.69	31,913	99.02			32,101	99.65	31,898	99.02		
Yes	170	0.31	317	0.98			114	0.35	317	0.98		
MDD ^3^					−0.06	<0.001					−0.05	<0.001
No	54,660	98.76	31,579	97.98			31,773	98.63	31,564	97.98		
Yes	685	1.24	651	2.02			442	1.37	651	2.02		
CKD ^3^					−0.13	<0.001					−0.08	<0.001
No	51,169	92.45	28,540	88.55			29,312	90.99	28,532	88.57		
Yes	4176	7.55	3690	11.45			2903	9.01	3683	11.43		
Asthma					−0.14	<0.001					−0.11	<0.001
No	53,685	97.00	30,302	94.02			31,048	96.38	30,287	94.02		
Yes	1660	3.00	1928	5.98			1167	3.62	1928	5.98		
COPD ^3^					−0.19	<0.001					−0.15	<0.001
No	53,077	95.90	29,448	91.37			30,653	95.15	29,434	91.37		
Yes	2268	4.10	2782	8.63			1562	4.85	2781	8.63		
HF ^3^					−0.08	<0.001					−0.05	<0.001
No	54,226	97.98	31,150	96.65			31,429	97.56	31,139	96.66		
Yes	1119	2.02	1080	3.35			786	2.44	1076	3.34		
URI ^3^					−0.63	<0.001					−0.59	<0.001
No	39,636	71.62	13,536	42.00			22,565	70.05	13,527	41.99		
Yes	15,709	28.38	18,694	58.00			9650	29.95	18,688	58.01		
GERD ^3^					−0.12	<0.001					−0.11	<0.001
No	54,765	98.95	31,368	97.33			31,849	98.86	31,353	97.32		
Yes	580	1.05	862	2.67			366	1.14	862	2.68		
Epilepsy					−0.02	0.004					−0.01	0.075
No	55,199	99.74	32,109	99.62			32,121	99.71	32,095	99.63		
Yes	146	0.26	121	0.38			94	0.29	120	0.37		

^1^ Chi-square test. ^2^ The matching variables and the unit of insured salary are in New Taiwan dollars (NTD). ^3^ Abbreviations: SMD, Standardized mean difference; CCI, Charlson comorbidity index; DM, diabetes mellitus; AD, Alzheimer’s disease; PD, Parkinson’s disease; MDD, major depression disorder; CKD, chronic kidney disease; COPD, chronic obstructive pulmonary disease; HF, heart failure; URI, upper respiratory infection; GERD, gastroesophageal reflux disease.

**Table 3:** The incidence rate of pneumonia in elderly patients.

Variables	Pneumonia		Risk Ratio
	N	Incident Rate (‰)	
Total	1519	23.58	
Anticholinergic drugs			
Without	529	15.27	
With	990	33.23	2.18
Diabetes mellitus			
No	1036	21.82	
Yes	483	28.48	1.31
Alzheimer’s disease			
No	1507	23.52	
Yes	12	33.61	1.43
Stroke			
No	1215	20.99	
Yes	304	46.48	2.21
Parkinson’s disease			
No	1490	23.28	
Yes	29	67.29	2.89
Major depression disorder			
No	1493	23.57	
Yes	26	23.79	1.01
Chronic kidney disease			
No	1255	21.70	
Yes	264	40.09	1.85
Asthma			
No	1187	19.35	
Yes	332	107.27	5.54
Chronic obstructive pulmonary disease			
No	1020	16.98	
Yes	499	114.90	6.77
Heart failure			
No	1404	22.44	
Yes	115	61.76	2.75
Upper respiratory infection			
No	594	16.46	
Yes	925	32.64	1.98
Gastroesophageal reflux disease			
No	1458	23.07	
Yes	61	49.67	2.15
Epilepsy			
No	1496	23.30	
Yes	23	107.48	4.61

**Table 4:** Comparing the risk of incident pneumonia between elderly patients with and without anticholinergic drugs.

Variables	aOR ^1,2^	95% Confidence Interval		*p*-Value
Anticholinergic agent	1.33	1.18–1.49	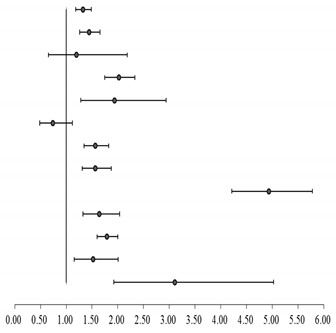	<0.001
DM ^1^	1.44	1.26–1.65		<0.001
AD ^1^	1.20	0.66–2.18		0.561
Stroke	2.02	1.75–2.33		<0.001
PD ^1^	1.94	1.28–2.94		0.002
MDD ^1^	0.74	0.49–1.12		0.158
CKD ^1^	1.57	1.35–1.82		<0.001
Asthma	1.57	1.31–1.87		<0.001
COPD ^1^	4.93	4.21–5.78		<0.001
HF ^1^	1.64	1.32–2.04		<0.001
URI ^1^	1.79	1.60–2.00		<0.001
GERD ^1^	1.52	1.15–2.01		0.003
Epilepsy	3.11	1.92–5.03		<0.001

^1^ Abbreviations: aOR, adjusted odds ratio; DM, diabetes mellitus; AD, Alzheimer’s disease; PD, Parkinson’s disease; MDD, major depression disorder; CKD, chronic kidney disease; COPD, chronic obstructive pulmonary disease; HF, heart failure; URI, upper respiratory infection; GERD, gastroesophageal reflux disease. ^2^ The conditional logistic regression model, stratified by matching variables, was used to estimate the risk.

## Appendix A

**Table A1:** The list of anticholinergic drugs.

Anticholinergic Drugs
Amitriptyline hydrochloride
Atropine products
Benztropine mesylate
Carisoprodol
Chlorpheniramine maleate
Chlorpromazine hydrochloride
Cyproheptadine hydrochloride
Dicyclomine hydrochloride
Diphenhydramine hydrochloride
Fluphenazine hydrochloride
Hydroxyzine hydrochloride and hydroxyzine pamoate
Hyoscyamine products
Imipramine hydrochloride
Meclizine hydrochloride
Oxybutynin chloride
Perphenazine
Promethazine hydrochloride
Thioridazine hydrochloride
Thiothixene
Tizanidine hydrochloride
Trifluoperazine hydrochloride
Amantadine hydrochloride
Baclofen
Cetirizine hydrochloride
Cimetidine
Clozapine
Cyclobenzaprine hydrochloride
Desipramine hydrochloride
Loperamide hydrochloride
Loratadine
Nortriptyline hydrochloride
Olanzapine
Prochlorperazine maleate
Pseudoephedrine hydrochloride-triprolidine hydrochloride
Tolterodine tartrate
Carbidopa-levodopa
Entacapone
Haloperidol
Metocarbamol
Metoclopramide hydrochloride
Mirtazapine
Paroxetine hydrochloride
Pramipexole dihydrochloride
Quetiapine fumarate
Ranitidine hydrochloride
Risperidone
Selegiline hydrochloride
Trazodone hydrochloride
Ziprasidone hydrochloride

Source: Rudolph, J.L.; Salow, M.J.; Angelini, M.C.; McGlinchey, R.E. The anticholinergic risk scale and anticholinergic adverse effects in older persons. *Arch. Intern. Med.* **2008**, *168*, 508–513.
